# Lifestyle Changes, Emotional Eating, Gender, and Stress during COVID-19 Lockdown

**DOI:** 10.3390/nu14183868

**Published:** 2022-09-18

**Authors:** Dorit Hadar-Shoval, Michal Alon-Tirosh, Kfir Asraf, Lubna Tannous-Haddad, Orna Tzischinsky

**Affiliations:** 1 Psychology Department, The Max Stern Yezreel Valley College, Yezreel Valley 1930600, Israel; 2 Behavioral Sciences Department, The Max Stern Yezreel Valley College, Yezreel Valley 1930600, Israel; 3 Educational Counseling Department, The Max Stern Yezreel Valley College, Yezreel Valley 1930600, Israel

**Keywords:** emotional eating, stress, eating habits, physical activity, alcohol consumption, cigarette smoking, sleep quality, nature and magnitude of change

## Abstract

Emotional eating poses health risks. It is associated with adverse weight gain and a higher body mass index and is frequently triggered by stressful situations such as pandemics. The COVID-19 pandemic was found to cause stress as well as lifestyle changes of different magnitudes. This study examined the relationship between lifestyle changes and emotional eating by focusing on the moderating effect of gender and COVID-19-related stressors. A total of 1969 respondents completed online questionnaires during the first COVID-19 lockdown in Israel. The questionnaires assessed COVID-19-related lifestyle changes concerning eating habits, alcohol consumption, sleep quality, physical activity, and cigarette smoking, COVID-19-related stressors, and emotional eating (Dutch Eating Behavior Questionnaire). People with positive and negative COVID-19-related lifestyle changes demonstrated higher emotional eating compared to people with no lifestyle changes. The relationship between lifestyle changes and emotional eating was moderated by gender and COVID-19-related stressors. In conclusion, health officials should consider recommendations about lifestyle changes given to the public in times of unpredictable changes, with special attention to populations at risk of emotional eating. As emotional eating is related to poor emotional regulation skills, public nutrition recommendations should focus on improving these skills rather than adopting better diets.

## 1. Introduction

The COVID-19 pandemic and its accompanying conditions (such as social distancing, quarantine, lockdowns, and occupational changes) have had unexpected and unpredictable consequences for most people around the globe. The pandemic has led to abrupt changes in the way that people live, work, study, play, exercise, interact, and eat [1,2,3,4]. Life events literature indicates that unpredictable changes may have an impact on emotional regulation, namely, the ability to regulate and control one’s emotions. This is an important psychological skill that is linked to good mental and physical health [5]. One manifestation of emotional regulation is emotional eating, which is an attempt to control negative emotions through eating [6]. This study aims to learn about the relationship between lifestyle changes due to COVID-19 and emotional eating and the moderating effect of gender and perceived COVID-19 stress in this relationship.

### 1.1. Lifestyle Changes

One of the most adopted health policies during the COVID-19 pandemic was social distancing, which was accompanied by restrictions on face-to-face activities and participation in public services. These restrictions led to changes in people’s lifestyles and daily conduct at home and in social contexts. Significant changes were noted in lifestyle habits such as physical activity, sleeping habits, cigarette smoking, alcohol consumption, and eating habits [7] concerning both the nature and the magnitude of change. Studies have reported that some people changed their lifestyle habits negatively, meaning they adopted unhealthy behaviors such as excessive cigarette smoking, reduction in physical activity, and increased consumption of unhealthy foods [8,9,10]. Other studies have addressed positive changes and the adoption of healthier behaviors, such as increasing physical activity or switching to more balanced or plant-derived nutrition such as the Mediterranean diet [9,11].

The research literature has paid more attention to the negative changes resulting from the pandemic, but positive changes should also be investigated as both kinds of changes were found to be linked to stress and high psychological distress throughout [7]. These findings are in line with psychological literature indicating that all life events that include changes, regardless of their nature (negative or positive), influence mental health and are associated with poor emotional regulation and psychological distress [12,13,14].

The studies demonstrate that unexpected and unpredictable changes are shown to have an effect on emotional regulation, which is linked to feelings of distress. Emotional regulation refers to an individual’s ability to regulate emotions, namely, to shape the emotions they feel and control how they experience and express them [15]. This ability was found to be connected to good mental and physical health [5].

Life events studies examining emotional regulation found links between unpredictable life events in childhood and reduced emotional regulation in adulthood [14,15,16,17]. The studies suggest that unpredictability is likely to have a strong association with emotional control difficulties.

The COVID-19 pandemic and its accompanying conditions were mainly characterized by their unpredictability and not necessarily by their severity or harshness. For example, in Israel, in the first weeks of the pandemic, death rates were low, and the pandemic was mainly felt through the numerous unpredictable lifestyle changes it caused (due to lockdown and social distancing). Though some of the changes were positive in nature (e.g., the ability to spend more time with family, engage in physical activity, and eat more regularly), studies have indicated increased psychological distress among the Israeli public at that time [18], reflected in, among other things, emotional eating, which is an expression of difficulty in emotional regulation [6].

### 1.2. Emotional Eating

Emotional eating means the overconsumption of food as a response to negative emotions and stress as a possible way of regulating emotions [6]. Unpredictable changes, which cause stress and psychological distress, can often be a trigger for emotional eating [19]. Studies have found a high degree of emotional eating among people with poor emotional regulation skills [6,20,21]. For example, a study conducted among Chinese adolescents found an association between emotional regulation strategies (suppression/cognitive reappraisal) and an energy-rich diet in girls [22]. The study also provided evidence that higher levels of suppression may put girls at risk of emotional eating, potentially affecting their energy-rich dietary patterns. In another study, emotional regulation was related to both emotional eating and dietary restraint [23].

Research evidence has suggested that emotional eating is usually characterized by people preferring particularly tasty foods (e.g., foods high in calories, fat, and/or sugar) [6,24] which are not consistent with good health [25]. Emotional eating poses a health risk as it is associated with adverse weight gain and a higher body mass index (BMI) [26]. During the COVID-19 pandemic, research from different countries reported increased emotional eating [27]. For example, a study conducted in Saudi Arabia among healthy young women found that emotional eating was common during the pandemic, with almost one in two women identified as emotional eaters [28]. In addition, some studies found links between emotional eating and mental health aspects. For example, a study conducted in Norway found that psychological distress was strongly associated with emotional eating [6]. Likewise, in a study conducted in the United States, perceived stress was found to be significantly correlated with emotional eating [29].

### 1.3. Moderating Role of Gender and Perceived COVID-19 Stress

COVID-19 studies have suggested that the pandemic and its accompanying conditions, which caused unpredictable changes in lifestyle habits, are associated with a decrease in emotional regulation and an increase in emotional eating among the general population; however, some populations have been found to be more prone to emotional eating than others.

Studies prior to the COVID-19 pandemic have indicated some at-risk populations who consume more energy-rich and sweet snack foods than others even under normal circumstances and who are therefore likely to increase their emotional eating in times of stress [30]. Two populations were identified as being at particular risk of emotional eating: people who perceive situations in a more stressful way [3] and women [25]. The tendency toward emotional eating when encountering negative emotions and stress was found even stronger among emotional eaters [6,24], and stress-induced emotional eating was found to be more common among women than men [31]. These at-risk populations should receive special attention in both research and practice.

### 1.4. The Current Study

**Figure 1:**
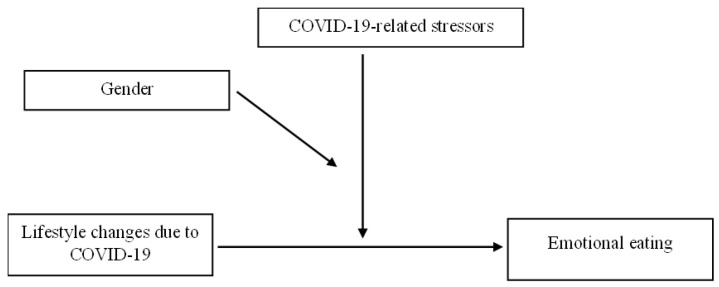
The moderating effect of gender and COVID-19-related stressors on the relationship between lifestyle changes due to COVID-19 and emotional eating.

There are existing studies addressing both lifestyle changes, specifically, eating habits, and emotional eating during the COVID-19 pandemic. However, there remains a need for a deeper examination of the relationship between them which should pay attention to special at-risk populations and address the nature and magnitude of the lifestyle changes. The current study examines these issues by focusing on changes in five health-related behaviors: eating habits, alcohol consumption, physical activity, sleep quality, and cigarette smoking. It has two main hypotheses. The first hypothesis expects a difference in emotional eating between individuals who experienced lifestyle changes (both positive and negative) due to COVID-19 and individuals who did not experience such changes. The second hypothesis expects the relationship between lifestyle changes due to COVID-19 and emotional eating to be moderated by gender and COVID-19-related stressors (Figure 1).

## 2. Materials and Methods

### 2.1. Participants and Data Collection

The study used data collected in a cross-sectional survey administered during the first COVID-19 lockdown in Israel. The survey assessed the public’s mental health by investigating psychological and behavioral responses during the pandemic. An anonymous questionnaire was sent online using iPanel (https://www.ipanel.co.il accessed on 9 August 2022), a large Israeli panel service. Inclusion criteria included being between 18 and 75 years of age and speaking the language in which the questionnaire was administered (either Hebrew or Arabic). No exclusion criteria were applied. Questionnaire completion was voluntary, and respondents were told that they could stop their participation at any point. Participants who completed the questionnaire were excluded from the final analysis (1% of completed questionnaires) if they failed attention checks or completed the measures in less than 10 min and if their responses were implausible (e.g., they chose the same answer throughout the questionnaire). The final analysis included 1969 participants. Table 1 presents their demographic data.

### 2.2. Measures

#### 2.2.1. Demographic Questionnaire

The participants reported their gender, age, income status, years of education, and work status since the onset of the pandemic. They also answered six questions about their eating behaviors, ranking the frequency of each eating behavior since the beginning of the lockdown between 1 (never) and 5 (very frequently). Behaviors included: eating at the same time every day, eating with a family member, eating more than before, being satisfied with your eating habits, eating unhealthy food, and feeling that you are keeping a balanced and healthy diet.

#### 2.2.2. Lifestyle Changes Due to COVID-19

We measure lifestyle changes due to COVID-19 via two questions relating to the nature and magnitude of change for each of the lifestyle habits. This methodology has been used in previous studies [7,32,33,34]. Table 2 presents the questions and the possible answers regarding the nature of change (full details on this measure can be found in [7]). In order to measure the magnitude of change, participants indicated whether there was no change (0), a moderate change (1), or substantial change (2) in each of the lifestyle habits. The magnitude of change score was calculated as the sum of the scores of all lifestyle change variables (ranging from 0–10).

#### 2.2.3. Emotional Eating

Emotional eating was measured using a 13-item version of the Dutch Eating Behavior Questionnaire (DEBQ) emotional eating scale (Hebrew version) [35,36]. Items were rated on a 5-point Likert scale ranging from never [1] to very often [5]. The emotional eating score was calculated as the mean score of the 13 items. Higher total scores indicate a greater tendency to engage in emotional eating. Internal reliability (Cronbach’s alpha) was 0.95. 

#### 2.2.4. COVID-19-Related Stressors

COVID-19-related stressors were calculated as the sum of the stress reported regarding 13 COVID-19-related stressors. This methodology has been used in previous studies [2,37,38]. Higher scores indicate greater stress. Internal consistency (Cronbach’s alpha) was 0.87.

### 2.3. Statistical Analysis

Normality of the study’s variables was examined via skewness and kurtosis values; none of them exceeded |2|. Group differences in emotional eating (DEBQ total score) in the lifestyle change variables were assessed using Welch’s one-way ANOVA, with Games-Howell post hoc tests and bootstrapped 95% confidence intervals (bias-corrected and accelerated, 5000 samples) for pairwise comparisons. Moderation analysis was conducted using Model 3 of Hayes’ PROCESS macro (v 4.0) for SPSS [39]. The predictors were lifestyle changes, gender, and COVID-19-related stressors. Covariates in the model were age, family status, work status during the pandemic, place of residence, and income status since the onset of the pandemic. The dependent variable was emotional eating. Bootstrap-derived 95% CIs were reported for the coefficients. Data deviating more than |2.5| SD from the group mean were considered statistical outliers and were excluded from all analyses.

## 3. Results

### 3.1. Descriptive Statistics of the Research Variables

The mean DEBQ total score was 2.33 ± 1.06 (range: 1–5). The mean magnitude of change was 3.24 ± 1.79, and the median was 3 (range: 0–10), indicating that more than half of the participants experienced a change in more than two lifestyle habits at different magnitudes. The mean of COVID-19-related stressors was 32.75 ± 8.11, and the median was 32 (range: 13–52). Table 3 displays the descriptive statistics of the research variables.

Table 4 presents the descriptive statistics of the participants’ eating behavior. During COVID-19, most of the participants maintained a regular meal schedule (52.78%) and ate together with other family members (49.51%). Additionally, a large proportion of the cohort reported eating more than before the onset of the pandemic (36.28%), feeling dissatisfied with their eating habits (41.74%), eating unhealthy food (31.68%), and feeling they were not maintaining a balanced and healthy diet (41.95%).

### 3.2. Relationship between the Nature of Lifestyle Changes Due to COVID-19 and Emotional Eating

The means and standard deviations of emotional eating in each change condition (positive, negative, and no change) and in each lifestyle variable (eating habits, alcohol consumption, physical activity, sleep quality, and cigarette smoking) are presented in Table 5.

#### 3.2.1. Eating Habits

The ANOVA was statistically significant with a large effect size. Post hoc analyses demonstrated that the no change group showed significantly less emotional eating than the positive change group (*p* < 0.001, CI = −0.97, −0.81, Hedges’ *g* = 1.12) and the negative change group (*p* < 0.001, CI = −1.23, −1.03, Hedges’ *g* = 1.18). The group with the positive change reported significantly less emotional eating than the negative change group (*p* < 0.001, CI = −0.34, −0.13, Hedges’ *g* = 0.23).

#### 3.2.2. Alcohol Consumption

The ANOVA was statistically significant with a small effect size. Post hoc analyses demonstrated that the no change group reported significantly less emotional eating than the negative change group (*p* < 0.001, CI = −0.61, −0.25, Hedges’ *g* = 0.47) but not when compared to the positive change group (*p* = 0.154, CI = −0.30, 0.01, Hedges’ *g* = 0.17). The positive change group reported significantly less emotional eating than the negative change group (*p* = 0.019, CI = −0.49, −0.08, Hedges’ *g* = 0.30).

#### 3.2.3. Physical Activity

The ANOVA was statistically significant with a small effect size. Post hoc analyses demonstrated that the no change group reported significantly less emotional eating than the positive change group (*p* = 0.001, CI = −0.40, −0.11, Hedges’ *g* = 0.28) and the negative change group (*p* < 0.001, CI = −0.48, −0.24, Hedges’ *g* = 0.35). The positive and negative groups were not significantly different from each other (*p* = 0.274, CI = −0.24, 0.02, Hedges’ *g* = 0.10).

#### 3.2.4. Sleep Quality

The ANOVA was statistically significant with a small effect size. Post hoc analyses demonstrated that the no change group reported significantly less emotional eating than the positive change group (*p* < 0.001, CI = −0.43, −0.23, Hedges’ *g* = 0.33) and the negative change group (*p* < 0.001, CI = −0.87, −0.33, Hedges’ *g* = 0.61). The positive change group reported significantly less emotional eating than the negative change group (*p* = 0.113, CI = −0.52, −0.002, Hedges’ *g* = 0.24). The bootstrap-derived CI of this comparison does not include zero, which suggests that the comparison is statistically significant.

#### 3.2.5. Cigarette Smoking

The ANOVA was statistically significant with a small effect size. Post hoc analyses demonstrated that the no change group reported significantly higher emotional eating than the positive change group (*p* = 0.006, CI = 0.08, 0.40, Hedges’ *g* = 0.25) but significantly lower emotional eating than the negative change group (*p* = 0.005, CI = −0.41, −0.09, Hedges’ *g* = 0.25). The positive change group reported significantly less emotional eating than the negative change group (*p* < 0.001, CI = −0.70, −0.28, Hedges’ *g* = 0.48).

### 3.3. The Moderating Effect of Gender and COVID-19-Related Stressors on the Relationship between the Magnitude of Change and Emotional Eating

Prior to the moderation analysis, we first calculated Pearson’s correlations to examine the correlations between the variables (Table 6). All the correlations were positive and statistically significant with small but robust effects.

**Figure 2:**
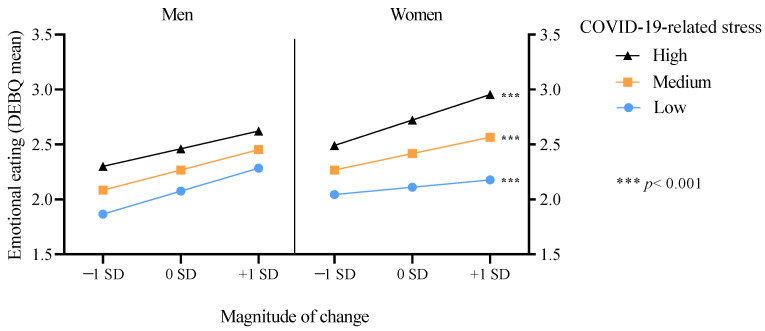
Moderating effect of COVID-19-related stress and gender on the relationship between the magnitude of change and emotional eating.

Subsequently, we examined whether gender and COVID-19-related stressors moderated the relationship between lifestyle changes due to COVID-19 and emotional eating. The model was significant (*F*(12, 1595) = 20.49, *p* < 0.001) and explained 13.36% of the variance (R^2^). The three-way interaction term (gender X COVID-19-related stressors X magnitude of change) was statistically significant (B = −0.003, *p* = 0.032, CI = −0.0072, −0.0001). Probing the interaction, we examined whether the COVID-19-related stressors X magnitude of the change interaction is different between men and women. While the interaction was significant for women (B = 0.005, *F*(1, 1595) = 5.29, *p* = 0.021), it was not for men (B = −0.001, *F*(1, 1595) = 0.48, *p* = 0.487). Regarding women, the two independent variables were split into three conditions: (−1 SD) low, (0 SD) medium, and (+1 SD) high. The slopes of all conditions of the moderator (COVID-19-related stressors) were statistically significant, and hence the relationships between emotional eating and magnitude of change in the low (B = 0.12, t = 4.05, *p* < 0.001), medium (B = 0.17, t = 8.08, *p* < 0.001), and high (B = 0.21, t = 7.96, *p* < 0.001) categories were all different from zero. The trajectory was similar for all conditions, i.e., the higher the magnitude of change, the greater the emotional eating. However, this effect was the strongest when the COVID-19-related stressors score was also high (Figure 2).

## 4. Discussion

The COVID-19 pandemic has led to unpredictable and abrupt changes in people’s lives globally. Lifestyle habits, such as physical activity, sleep quality, cigarette smoking, alcohol consumption, and eating habits, have significantly changed [7,33], and studies have reported an increase in stress and its accompanying consequences, such as emotional eating [3,7,13]. In the current study, more than one-third of the participants reported a change in their eating behaviors since the beginning of the COVID-19 lockdown, and half of the participants reported an emotional eating score higher than 2.15 (on a 1–5 scale). The study also found that most of the participants reported more than two changes in their lifestyle habits. These findings, which imply significant changes in lifestyle habits as well as a relatively high prevalence of emotional eating, are consistent with findings of other studies conducted during the pandemic. For example, a high prevalence of emotional eating was also found in studies conducted in Saudi Arabia and Norway [6,28]. Significant changes in lifestyle habits were reported in studies conducted in Australia, Spain, as well as in a comparative worldwide study [9,10,33].

The study examined the relationship between lifestyle changes and emotional eating. Its findings support the first hypothesis that emotional eating differs between individuals who experienced changes in lifestyle habits and individuals who did not experience such changes, with the former demonstrating increased emotional eating. Although there is an indication that individuals who experienced negative lifestyle changes exhibited more emotional eating than those who experienced positive changes, both kinds of changes were associated with high emotional eating. These findings are in line with life events studies indicating that unpredictable changes (regardless of their nature) are associated with stress [12,13] and linked to decreased emotional regulation [14]. Decreased emotional regulation is associated with emotional eating [20,21] as a possible way of regulating negative emotions [8].

Understanding that positive changes are also linked to emotional eating is important when considering nutrition recommendations given to the public during the pandemic. While several studies have discussed the benefits of adopting healthy diets during lockdowns [11,28,29], the current study considers the feasibility of dietary changes during this period by emphasizing the links between positive changes and emotional eating. Though it is possible that positive nutrition changes have long-term benefits, the current findings suggest that in situations involving unpredictable changes which cause stress, such as the pandemic and lockdown, all changes are associated with increased emotional eating regardless of their nature. 

In accordance with the second hypothesis, the findings indicate that both the level of COVID-19-related stressors and gender serve as moderating factors in the relationship between lifestyle changes and emotional eating. This relationship was found to be stronger among women who perceived the pandemic, lockdown, and social distancing regulations as more stressful. These findings are consistent with previous studies showing that both women and people who tend to perceive situations as stressful are more prone to emotional eating [3,17]. The study also highlights the increased risk among individuals belonging to both populations.

These findings are important both on a theoretical and clinical level. Theoretically, they allow us to better understand the mechanisms behind the relationship between unpredictable lifestyle changes and emotional eating and identify populations at risk. Clinically, these findings point to both the complexity of the situation and its possible solution. In other words, the finding that perceived COVID-19 stress moderates the relationship between lifestyle changes and emotional eating suggests that attempts to reduce emotional eating should be based on knowledge related to emotion regulation treatment strategies. Several strategies, such as mindfulness [40], acceptance and commitment therapy (ACT) [41], cognitive behavior therapy (CBT) [42], and dialectical behavior therapy (DBT) [43], have been found to be efficacious in reducing emotional eating and could be applied for the treatment of individuals. Yet, in a pandemic situation, it is more difficult to provide individual treatment to all those who need it. Therefore, these emotional regulation treatment strategies can serve as the basis for general guidelines to the public regarding nutrition. These guidelines should not focus on changing the eating behaviors of the public since this may prompt people who find it difficult to follow them to experience feelings of guilt and other negative feelings, which could, in turn, reinforce the negative feelings created by the pandemic. Instead, the guidelines should focus on the way people perceive the events and the negative feelings that accompany these perceptions and make suggestions regarding how to reduce stress and worry, with specific reference to at-risk populations.

The finding that women, especially women who perceive events as stressful, are at high risk for emotional eating emphasizes the importance of considering gender aspects when making public health recommendations. Gender aspects should be considered in general health guidelines and in guidelines regarding nutrition in particular.

The current study’s cross-sectional design allowed for a large sample, the inclusion of multiple changes in lifestyle habits, and data collection in close proximity to the onset of the COVID-19 pandemic in Israel. Yet, its cross-sectional design is also responsible for several limitations. First, causality cannot be inferred. Second, it lacks information about the participants’ emotional eating over time (e.g., their emotional eating before the onset of the pandemic or any changes over time) and only represents their emotional eating at the time of data collection. 

## 5. Conclusions

As lifestyle changes (positive and negative) were found to be associated with increases in emotional eating, health officials should carefully consider recommendations about lifestyle changes given to the public in times of unpredictable changes, such as the COVID-19 pandemic. There should be special consideration for populations at risk of emotional eating, such as people who perceive situations in a more stressful way and women. As emotional eating is related to poor emotional regulation of stress skills, which reflects the individual’s mental health, this study highlights the role of nutritionists in emphasizing the maintenance of mental health as part as their recommendations for adopting better diets.

## Figures and Tables

**Table 1:** Participants’ demographics.

Variable	Descriptive Statistics
Gender	Male—44.89% (*n* = 884) Female—55.10% (*n* = 1085)
Age	40.40 ± 13.76 (20–75)
Family status	Married—62.87% (*n* = 1238) Not married—37.12% (*n* = 731)
Years of education	14.62 ± 2.50 (10–21)
Place of residence	Urban—75.06% (*n* = 1478) Rural—24.93% (*n* = 491)
Work status during the pandemic	Kept working—74.43% (*n* = 1217) Furloughed/Fired—25.56% (*n* = 418)
Income status since the onset of the pandemic	Not affected—35.24% (*n* = 694) Moderately affected—33.87% (*n* = 667) Severely affected—30.87% (*n* = 608)

Note: Continuous variables are presented as the mean ± SD (range), and categorical data as % (*n*).

**Table 2:** Questions and possible answers regarding the nature of change.

Questions	Possible Answers		
Since the outbreak of COVID-19 and the beginning of quarantines and lockdowns…	Positive Change	Negative Change	No Change
Have your eating habits changed?	I started eating a healthier diet	I started eating unhealthier food	I have not changed my eating habits
Have your alcohol consumption changed?	I started consuming alcohol in a more balanced way	I have increased my alcohol consumption	I have not changed my alcohol consumption
Have your physical activity habits changed?	I exercise more	I exercise less	I have not changed my physical activity
Has your sleep quality changed?	My sleep quality has improved	My sleep quality has deteriorated	My sleep quality has not changed
Have your smoking habits changed?	I smoke less cigarettes than I used to	I smoke more cigarettes than I used to	I have not changed the number of cigarettes I smoke

**Table 3:** Description of the study’s main variables.

Study’s Main Variables	Mean	SD	Median	Range
DEBQ total score	2.33	1.06	2.15	1–5
Magnitude of change	3.24	1.79	3	0–10
COVID-19-related stressors	32.75	8.11	32	13–52

**Table 4:** Description of the sample’s eating behaviors.

Eating Behavior	Never/Seldom	Sometimes	Frequently/Very Frequently
Eating at the same time every day	22.48% (*n* = 440)	24.73% (*n* = 484)	52.78% (*n* = 1033)
Eating with family member	21.15% (*n* = 414)	29.33% (*n* = 574)	49.51% (*n* = 969)
Eating more than before	38.06% (*n* = 745)	25.65% (*n* = 502)	36.28% (*n* = 710)
Satisfied with eating habits	41.74% (*n* = 817)	25.85% (*n* = 506)	32.39% (*n* = 634)
Eating unhealthy food	42.0% (*n* = 823)	26.26% (*n* = 514)	31.68% (*n* = 620)
Feeling you are maintaining balanced and healthy diet	41.95% (*n* = 821)	26.57% (*n* = 520)	31.47% (*n* = 616)

**Table 5:** Differences in emotional eating due to change in lifestyle variables.

	No Change	Positive Change	Negative Change	Welch’s *F* (*df*)	*p* (η^2^)
Eating habits	1.50 ± 0.57 *n* = 429	2.40 ± 0.88 *n* = 815	2.64 ± 1.13 *n* = 673	344.69 (2, 1217.71)	<0.001 (0.177)
Alcohol consumption	2.04 ± 0.82 *n* = 348	2.19 ± 0.83 *n* = 162	2.47 ± 1.04 *n* = 162	10.91 (2, 324.20)	<0.001 (0.037)
Physical activity	2.05 ± 0.85 *n* = 307	2.31 ± 0.97 *n* = 286	2.42 ± 1.08 *n* = 739	17.16 (2, 657.56)	<0.001 (0.021)
Sleep quality	2.12 ± 0.89 *n* = 515	2.46 ± 1.07 *n* = 1000	2.72 ± 1.34 *n* = 111	25.95 (2, 287.31)	<0.001 (0.029)
Cigarette smoking	2.25 ± 0.97 *n* = 1562	2.01 ± 0.89 *n* = 143	2.51 ± 1.10 *n* = 204	10.75 (2, 267.27)	<0.001 (0.011)

Note: Welch’s one-way ANOVA; data are presented as the mean ± SD.

**Table 6:** Pearson’s correlations between the study’s main variables.

N = 1969	DEBQ Total Score	Magnitude of Change	COVID-19-Related Stressors
Magnitude of change	0.309 *** (CI = 0.267, 0.350)	-	-
COVID-19-related stressors	0.237 *** (CI = 0.191, 0.283)	0.334 *** (CI = 0.295, 0.373)	-
Gender (1 = Female)	0.133 *** (CI = 0.090, 0.178)	0.075 *** (CI = 0.027, 0.121)	0.168 *** (CI = 0.124, 0.212)

*** *p* < *0*.001. Bootstrapped 95% BCa CI, 5000 samples. All correlations remained statistically significant after Bonferroni correction (*p* < 0.008).

## Data Availability

Data is available on request from the corresponding author.
